# rBMSC/Cav-1^F92A^ Mediates Oxidative Stress in PAH Rat by Regulating SelW/14-3-3*η* and CA1/Kininogen Signal Transduction

**DOI:** 10.1155/2019/6768571

**Published:** 2019-10-28

**Authors:** Wan-cheng Yu, Hai-ying Chen, Hong-li Yang, Peng Xia, Cheng-wei Zou, Tong-wen Sun, Le-xin Wang

**Affiliations:** ^1^Department of Cardiovascular Surgery, Shandong Provincial Hospital Affiliated to Shandong University, 324 Jingwu Road, Jinan, Shandong 250021, China; ^2^Central Laboratory of Liaocheng People's Hospital, Liaocheng, Shandong 252000, China; ^3^Department of Cardiology, Liaocheng People's Hospital and Affiliated Liaocheng People's Hospital of Shandong University, Liaocheng, Shandong 252000, China; ^4^Department of General ICU, The First Affiliated Hospital of Zhengzhou University, Henan Key Laboratory of Critical Care Medicine, Zhengzhou 450052, China; ^5^School of Biomedical Sciences, Charles Sturt University, Wagga Wagga, NSW 2650, Australia

## Abstract

**Background/Objectives:**

Carbonic anhydrase 1 (CA1)/kininogen and selenoprotein W (SelW)/14-3-3*η* signal transduction orchestrate oxidative stress, which can also be regulated by nitric oxide (NO). The mutated caveolin-1 (Cav-1^F92A^) gene may enhance NO production. This study explored the effect of Cav-1^F92A^-modified rat bone marrow mesenchymal stem cells (rBMSC/Cav-1^F92A^) on oxidative stress regulation through CA1/kininogen and SelW/14-3-3*η* signal transduction in a rat model of monocrotaline- (MCT-) induced pulmonary arterial hypertension (PAH).

**Method:**

PAH was induced in rats through the subcutaneous injection of MCT. Next, rBMSC/Vector (negative control), rBMSC/Cav-1, rBMSC/Cav-1^F92A^, or rBMSC/Cav-1^F92A^+L-NAME were administered to the rats. Changes in pulmonary hemodynamic and vascular morphometry and oxidative stress levels were evaluated. CA1/kininogen and SelW/14-3-3*η* signal transduction, endothelial nitric oxide synthase (eNOS) dimerization, and eNOS/NO/sGC/cGMP pathway changes were determined through real-time polymerase chain reaction, Western blot, or immunohistochemical analyses.

**Results:**

In MCT-induced PAH rats, rBMSC/Cav-1^F92A^ treatment reduced right ventricular systolic pressure, vascular stenosis, and oxidative stress; downregulated CA1/kininogen signal transduction; upregulated SelW/14-3-3*η* signal transduction; and reactivated the NO pathway.

**Conclusions:**

In a rat model of MCT-induced PAH, rBMSC/Cav-1^F92A^ reduced oxidative stress by regulating CA1/kininogen and SelW/14-3-3*η* signal transduction.

## 1. Background

Pulmonary arterial hypertension (PAH) is a pulmonary vascular disease that is associated with a high incidence of morbidity and mortality [1]. The treatment of PAH has been challenging, with vasodilating medications being the mainstays of therapy, although stem cell therapies have emerged as a promising future treatment [1–3]. One of the primary characteristics of PAH is pathological vascular remodeling [1–3]. In PAH, the remodeling of the distal pulmonary artery impedes the ejection of blood by the right ventricle, resulting in elevated pressure of the pulmonary artery that progresses to right ventricular failure [2]. Although the primary trigger of PAH remains incompletely understood, oxidative stress may have a crucial role in the development and progression of PAH [3].

Evidence for the participation of excessive oxidative stress in the pathogenesis of PAH is well-documented. Oxidative stress induces endothelial cell dysfunction and smooth muscle cell contraction that both contribute to PAH [4]. Moreover, oxidative stress triggers inflammatory processes within the vascular wall [5]; these processes are also involved in pulmonary injury [6]. Therefore, targeting excessive oxidative stress may advance PAH treatment [7]. Carbonic anhydrase 1 (CA1) and selenoprotein W (SelW) orchestrate various pathophysiological processes, including oxidative stress [8, 9]. CA1, a zinc-containing metalloenzyme, catalyzes the reversible hydration of carbon dioxide to protons (H^+^) and HCO_3_^−^ [10] and causes vascular injury by activating kininogen expression [11]. By contrast, SelW, the smallest selenoprotein that contains the canonical amino acid selenocysteine, protects cells against oxidative injury by upregulating 14-3-3*η* expression [8, 12, 13]. However, the change in CA1/kininogen and SelW/14-3-3*η* signal transduction in PAH has never been studied.

Novel PAH therapies based on mesenchymal stem cells (MSCs) have received increasing recognition given the high proliferative ability and multidirectional differentiation of MSCs [14]. In rat models of PAH, the MSC-based prostacyclin synthase gene attenuates pulmonary hypertension and improves prognosis [15]. Let-7a-modified MSCs ameliorate the progression of PAH and thus represent a promising therapeutic strategy for this disease [16]. We previously found that a mutated caveolin-1 (Cav-1^F92A^) gene that exhibits an alanine substitution for phenylalanine at position 92 modulates NO production in rat bone marrow mesenchymal stem cells (rBMSCs) [17]. Phenylalanine 92 (F92) is critical for the inhibitory actions of Cav-1 against endothelial nitric oxide synthase (eNOS), which inhibits NO production. The Cav-1^F92A^ gene can upregulate the activity of eNOS and enhance the production of NO [18], which performs diverse physiological actions, including antioxidation [19]. Dysfunctions in the NO pathway have been demonstrated in PAH [20]. Therefore, in the present study, we investigated whether rBMSC/Cav-1^F92A^ can mediate oxidative stress in rats with monocrotaline- (MCT-) induced PAH through the regulation of CA1/kininogen and SelW/14-3-3*η* signal transduction.

## 2. Methods

### 2.1. Animals

All experiments were approved by the Institutional Animal Care and Use Committee (Liaocheng People's Hospital, Shandong, China) and conducted in accordance with the “Guide for the Care and Use of Laboratory Animals” set by the National Institute of Health. Male Wistar rats (certificate number SCXK (Shandong) 20140007) with body weights of 125–150 g were obtained from the animal experimental center of Shandong University (Jinan, China). The rats were housed under a 12 h light/12 h dark cycle at 25 ± 1°C. Food and water were provided ad libitum.

### 2.2. Cell Isolation, Culture, Lentiviral Vector Packaging, and Transduction

rBMSC isolation, culture, lentiviral vector (LV) packaging, and transduction were all performed as previously described [17]. Briefly, rBMSCs (passage 3) in the exponential growth phase were randomly divided into five groups: control group, rBMSC/Vector group (transduced with pLVX-mCMV-mCherry lentivirus), rBMSC/Cav-1 group (transduced with LV-Cav-1 lentivirus), rBMSC/Cav-1^F92A^ group (transduced with LV-Cav-1^F92A^ lentivirus), and rBMSC/Cav-1^F92A^+L-NAME group (transduced with LV-Cav-1^F92A^ lentivirus and treated with L-NAME (2 mM, Beyotime Biotechnology, Jiangsu, China)). Transduction efficiency was observed under fluorescent microscopy (CKX71, Olympus) at 5 days post transduction.

### 2.3. PAH Model and Cell Transplantation

Rats received subcutaneous injections of MCT (60 mg/kg, Sigma Chemical Co., USA) for the construction of the PAH model. Rats that had been injected with 0.9% saline were set as the control group. After 14 days, rats that received MCT were randomly assigned to five groups (*n* = 10 in each group): rats treated with saline (model group), rats that received rBMSC/Vector (vector group), rats injected with rBMSC/Cav-1 (Cav-1 group), rats that received rBMSC/Cav-1^F92A^ (Cav-1^F92A^ group), and rats injected with rBMSC/Cav-1^F92A^+L-NAME (Cav-1^F92A^+L-NAME group). Approximately 1 × 10^6^ cells in saline were slowly injected into rats via the tail vein for cell transplantation.

### 2.4. Hemodynamic Evaluation and Pulmonary Vascular Morphometry

At 2 weeks after cell transplantation, the rats were subjected to invasive hemodynamic evaluation with a 2 Fr microtip catheter (Millar Instruments, Houston, TX). Animals were anesthetized with chloral hydrate (400 mg/kg, subcutaneous). The catheter was advanced into the right ventricle to obtain pressure measurements. Pulmonary artery pressure was estimated on the basis of right ventricular systolic pressure (RVSP). Each pressure reading was presented as the mean value of three measurements. Lungs were removed from the rats after euthanasia with an overdose of chloral hydrate (1.5 g/kg) and maintained in liquid N. The overdose of chloral hydrate resulted in rapid loss of consciousness followed by cardiac and respiratory arrest. Parts of the left lungs were extracted and fixed overnight in 10% neutral buffered formalin solution, embedded in paraffin, cut into sections with thicknesses of 5 *μ*m, and stained with hematoxylin and eosin (H&E). Paraffin-embedded sections were stained by using Masson's Trichrome Stain Kit (Beijing Solarbio Science & Technology Co., Ltd., Beijing, China) in accordance with the manufacturer's instructions. The percentage of wall area (WA%) of the total area was measured and calculated for the evaluation of the small pulmonary artery stenosis. Each experiment was repeated three times.

### 2.5. Measurement of Proinflammatory Cytokines, Reactive Oxygen Species, Glutathione, NO, and Tetrahydrobiopterin Levels

The effect of rBMSCs/Cav-1^F92A^ on the proinflammatory cytokine production was measured as previously described [21]. Briefly, the serum in each group was collected and measured using a flow cytometry analysis Cytometric Bead Array (CBA) Flex Set according to the BD CBA Rat Soluble Protein Master Buffer Kit (BD Biosciences PharMingen, San Diego, CA, USA) instruction manual. The concentration of interferon-*γ* (INF-*γ*), interleukin-1*α* (IL-1*α*), and tumor necrosis factor-*α* (TNF-*α*) was measured using flow cytometry. Each experiment was repeated for three times.

The levels of reactive oxygen species (ROS), glutathione (GSH), NO, and tetrahydrobiopterin (BH_4_) were quantified by using the Rat ROS ELISA Kit (Shanghai Xinfan Biotechnology Co., Ltd., Shanghai, China), Rat GSH ELISA Kit (Shanghai YuChun Biotechnology Co., Ltd., Shanghai, China), Nitric Oxide Colorimetric Assay Kit (BioVision, Milpitas, CA, USA), and Rat BH_4_ ELISA Kit (Shanghai Xinfan Biotechnology Co., Ltd., Shanghai, China), respectively, in accordance with the manufacturer's instructions. Data were obtained by using a Multiskan MK3 microplate reader at 540, 450, 450, and 450 nm. Each sample was analyzed in triplicate, and each experiment was repeated three times.

### 2.6. Immunohistochemistry

To visualize the presence of CA1 and SelW protein in lung tissues, paraffin-embedded samples were cut into sections with thicknesses of 5 *μ*m, deparaffinated, and hydrated. To quench endogenous peroxidase, samples were incubated for 15 min in 3% hydrogen peroxide at room temperature and then heated for 15 min in citrate buffer (pH 6.0) in a microwave oven at 90°C. Next, the samples were stained overnight with rabbit monoclonal anti-CA1 (1 : 200 dilution ratio, Bioworld Technology, Inc., Minnesota, USA) and anti-SelW (1 : 200 dilution ratio, Bioss, Massachusetts, USA) at 4°C. After 1 h of incubation with rabbit HRP-conjugated secondary antibodies (1 : 500 dilution ratio), sections were stained for 4 min by using a DAB Chromogen Substrate Kit (Maxin-Bio, Co., Fuzhou, China). Images were acquired under light microscopy (Olympus) and analyzed by using Image-Pro Plus 6.0 software.

### 2.7. Quantitative Polymerase Chain Reaction

The expression of guanosine-3′,5′-cyclic monophosphate (cGMP) mRNA in lung tissue was evaluated through quantitative polymerase chain reaction (qPCR). The frozen lung tissue was homogenized, and total RNA was extracted using a TRIzol reagent (TIANGEN Biotech Co., Ltd., Beijing, China). Then, 1 *μ*g of RNA was converted to cDNA and amplified using PrimeScript™ RT Master Mix (Takara Biotechnology Co., Ltd., Dalian, China). Real-time qPCR assays were performed in Applied Biosystems 7500 (ABI 7500, USA) using SYBR Premix Ex Taq™ (Takara Biotechnology Co., Ltd., Dalian, China). Relative gene expression was normalized to the expression of GAPDH, a housekeeping gene, through the 2^−*ΔΔ*CT^ method. Three independent replicates were performed to verify the reproducibility of the data. Primers were purchased from Sangon Biotech. The primer sequences are as follows: cGMP (sense: 5′-GCA GGC AAG ATT CAG AAC AAG TTG AC-3′ and antisense: 5′-GTG CTC GCT CCG CTG TAT GTA TG-3′) and GAPDH (sense: 5′-ATG ATT CTA CCC ACG GCA AG-3′ and antisense: 5′-CTG GAA GAT GGT GAT GGG TT-3′).

### 2.8. Western Blot Analysis

The frozen lung tissue was homogenized in ice cold lysis buffer containing PMSF (Beyotime Biotechnology, Jiangsu, China) and then centrifuged (5 min, 4°C, and 12000 g). The supernatant was then transferred to new tubes. Protein concentration was analyzed by using a BCA Protein Assay Kit (Beyotime Biotechnology, Jiangsu, China). Equal amounts of protein (15 *μ*g) were loaded onto 10% SDS-PAGE and transferred to a PVDF membrane (Millipore Corp., Billerica, MA, USA) through the wet transfer method. The membranes were then blocked for 1 h with 5% skimmed milk in TBST, then incubated overnight at 4°C with primary antibodies against eNOS (1 : 1000 dilution ratio, BD Biosciences, CA, USA), soluble guanylyl cyclase (sGC, 1 : 1000 dilution ratio, Abbiotec LLC, California, USA), CA1 (1 : 1000 dilution ratio, Bioworld Technology, Inc., Minnesota, USA), SelW (1 : 500 dilution ratio, Novus Biologicals, Colorado, USA), 14-3-3 protein (1 : 1000 dilution ratio, Abcam, Cambridge, United Kingdom), and kininogen (1 : 1000 dilution ratio, Abcam, Cambridge, United Kingdom). *β*-Actin (1 : 1000 dilution ratio, Beyotime Biotechnology, Jiangsu, China) served as the loading control. The PVDF membranes were incubated for 1 h with goat anti-rabbit or goat anti-mouse IgG/HRP secondary antibodies (1 : 1000 dilution ratio, Beyotime, Jiangsu, China). Protein bands were visualized using an ECL Western Blotting Kit (Beyotime Biotechnology, Jiangsu, China). The intensity of the resulting bands was measured by a densitometer and analyzed with AlphaView analysis software (ProteinSimple, USA).

### 2.9. Determination of the eNOS Dimer/Monomer Ratio

Lung tissue protein samples were not heated with Native Gel Sample Loading Buffer (4 : 1 ratio, Beyotime Biotechnology, Jiangsu, China). eNOS dimers and monomers were separated using Native-PAGE (Beyotime Biotechnology, Jiangsu, China), maintained at 4°C with Native-PAGE HEPES (Beyotime Biotechnology, Jiangsu, China) during electrophoresis, and transferred to a PVDF membrane (Millipore Corp., Billerica, MA, USA) using the wet transfer method. The PVDF membrane was blocked for 1 h with 5% skimmed milk in TBST at room temperature and incubated overnight at 4°C with primary antibodies against eNOS (1 : 1000 dilution ratio, BD Biosciences, CA, USA). Next, the membrane was incubated with goat anti-rabbit IgG/HRP secondary antibodies (1 : 1000 dilution ratio, Beyotime, Jiangsu, China) and analyzed by using AlphaView analysis software.

### 2.10. Statistical Analysis

Values were expressed as means ± SD. The statistical significance of differences was calculated through one-way ANOVA, followed by the SNK-*q* test using the SPSS 16.0 statistical package. *p* value < 0.05 was set as significant.

## 3. Result

### 3.1. Transduction Efficiency

**Figure 1:**
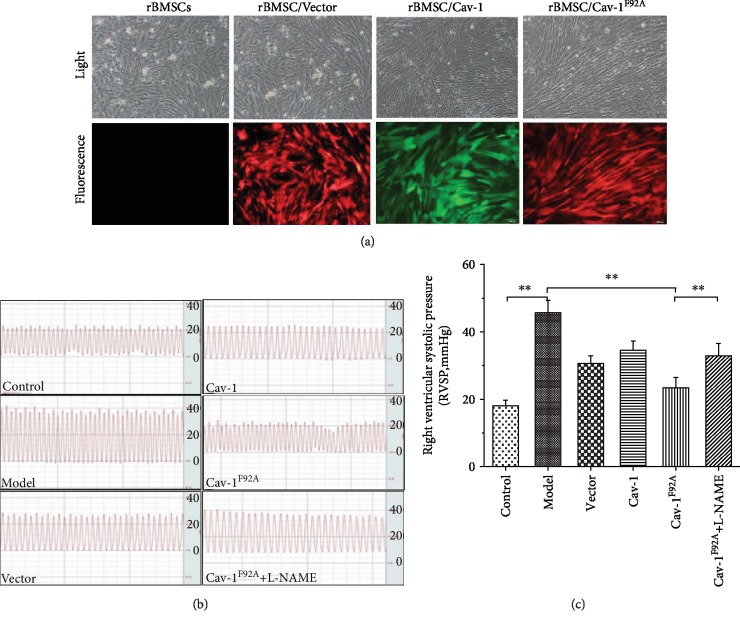
Transduction efficiency and the change of RVSP. (a) Lentiviral vector transduction efficiency at 5 days postinfection (bars = 100 *μ*m). (b, c) The effect of rBMSC/Cav-1^F92A^ on RVSP in MCT-induced PAH rats. RVSP: right ventricular systolic pressure. ^∗∗^*p* < 0.01.

Five days posttransduction, the green fluorescence protein expression was detected in Cav1 groups. The expression of red fluorescence protein was detected in negative groups and Cav-1^F92A^ groups. The transduction efficiency was greater than 80% in all groups (Figure 1(a)).

### 3.2. Effect of rBMSC/Cav-1^F92A^ on RVSP and Pulmonary Artery Stenosis

The RVSP of rats in the PAH groups increased (Figures 1(b) and 1(c)). The administration of rBMSC/Cav-1^F92A^ prevented the elevation of RVSP. Thisresult suggests that rBMSC/Cav-1^F92A^ decreases the pulmonary artery pressure of MCT-exposed rats (Figures 1(b) and 1(c)).

**Figure 2:**
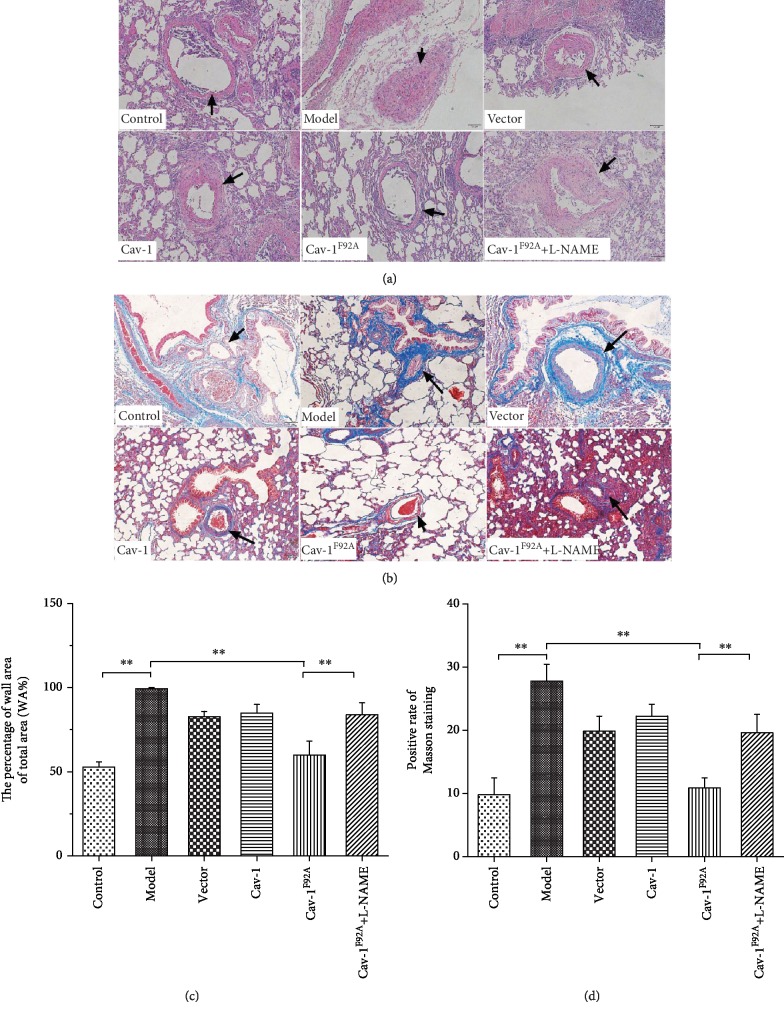
The effects of rBMSC/Cav1^F92A^ on pulmonary artery stenosis. Photomicrographs of serial sections of the peripheral rat lung containing small arteries from control animals or rats exposed to MCT. (a) H&E staining (bar = 50 *μ*m). (b) Masson staining. The staining showed the difference between different groups in the morphology of the small pulmonary arteries (bar = 50 *μ*m). (c) The percentage of the wall area of the total area (%). (d) The positive rate of Masson staining. The arrow indicates the pulmonary artery. ^∗∗^*p* < 0.01.

The pathological changes exhibited by the small pulmonary artery (100–300 *μ*m) were characterized through H&E and Masson staining. The small pulmonary artery in the control group presented a thin medial wall and large lumen. The wall thickness and small pulmonary artery stenosis of the PAH groups increased. The vascular morphology of rBMSC/Cav-1^F92A^-treated rats improved (Figures 2(a) and 2(c)). In PAH groups, Masson staining exhibited disorganized, proliferated collagen fibers within the vascular wall. These pathological changes improved under rBMSC/Cav-1^F92A^ treatment (Figures 2(b) and 2(d)). Consistent with the changes described previously, WA%, an indicator of small pulmonary artery stenosis, decreased in MCT-exposed rats under rBMSC/Cav-1^F92A^ treatment.

L-NAME, an eNOS inhibitor, abolished the inhibitory effect of rBMSC/Cav-1^F92A^ on RVSP and pulmonary artery stenosis (Figures 1(b) and 1(c) and Figure 2).

### 3.3. rBMSC/Cav-1^F92A^ Attenuates Inflammation and Oxidative Stress in the Lungs

**Figure 3:**
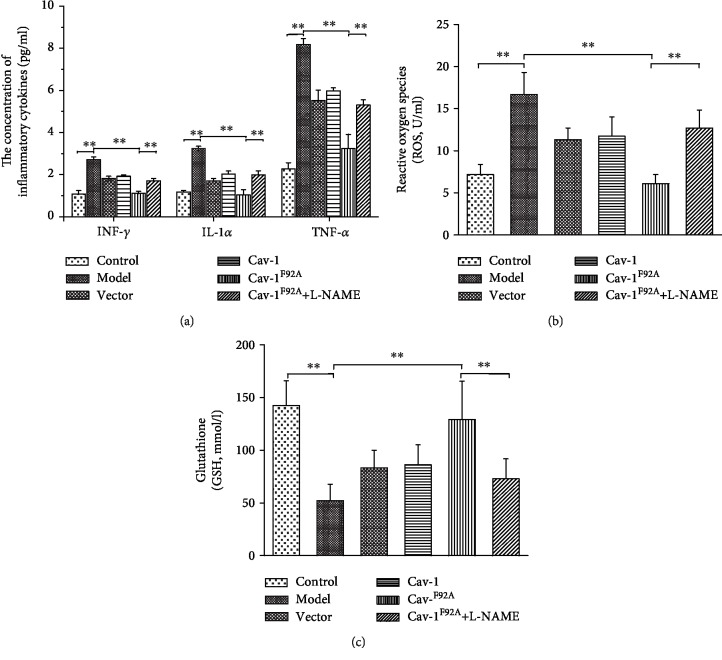
The effects of rBMSC/Cav1^F92A^ on inflammation and oxidative stress in PAH rats. (a) The level of proinflammatory cytokines INF-*γ*, IL-1*α*, and TNF-*α*. (b, c) The production of ROS and GSH. ^∗∗^*p* < 0.01.

Oxidative stress triggers the inflammatory response. Compared to the control groups, the proinflammatory cytokine levels of INF-*γ*, IL-1*α*, and TNF-*α* increased by approximately 2.5-, 2.6-, and 3.2-fold in MCT-exposed rats, respectively, and decreased under rBMSC/Cav-1^F92A^ treatment (Figure 3(a)).

ROS and GSH are commonly tested oxidative stress indices [22]. ROS increased approximately by 2.3-fold, and GSH decreased approximately by 0.36-fold in PAH groups relative to those in the control groups. rBMSC/Cav-1^F92A^ treatment attenuated oxidative stress, as demonstrated by the 0.36-fold decrease in ROS and 2.47-fold increase in GSH expression (Figures 3(b) and 3(c)).

### 3.4. Effect of rBMSC/Cav-1^F92A^ on the Expression of SelW, 14-3-3*η*, CA1, and Kininogen

**Figure 4:**
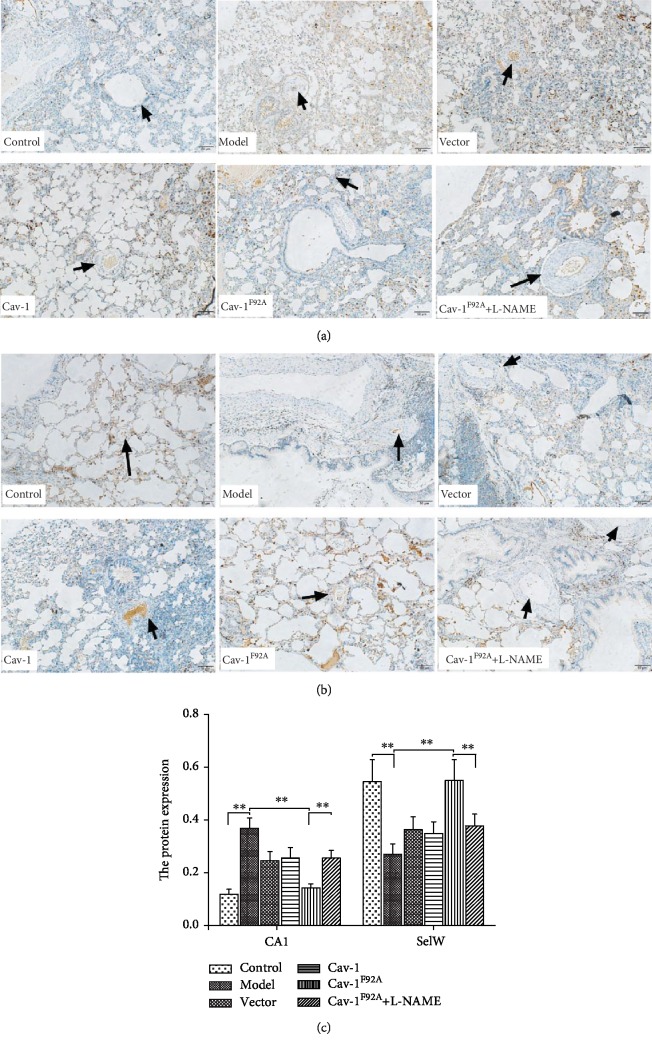
Immunohistology detection of CA1 and SelW expression in lung tissue. Photomicrographs of serial sections of the rat lung containing small arteries from control animals or rats exposed to MCT with saline vehicle or modulated rBMSCs. (a) Sections were immunostained for CA1 (bar = 50 *μ*m). (b) Sections were immunostained for SelW (bar = 50 *μ*m). (c) The protein of CA1 and SelW expression. The arrow indicates the pulmonary vascular. Positive expression is dark brown. ^∗∗^*p* < 0.01.

**Figure 5:**
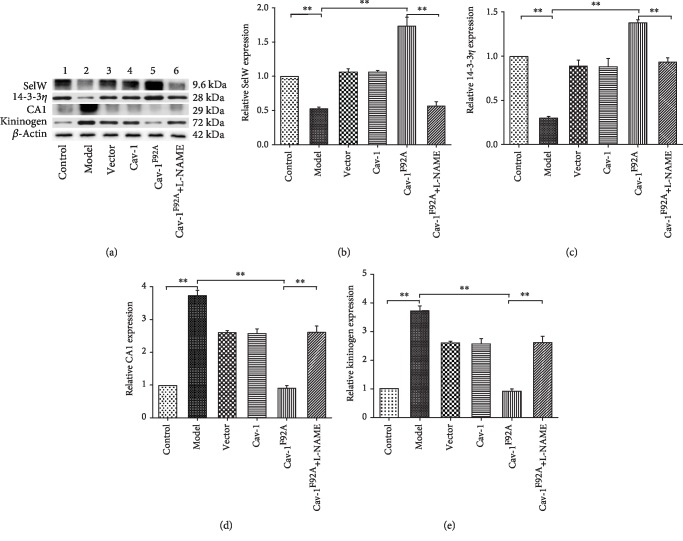
rBMSC/Cav1^F92A^ downregulated CA1/kininogen signal transduction and upregulated SelW/14-3-3*η* signal transduction. (a) Immunoblots of lung CA1, kininogen, SelW, and 14-3-3*η* expression in control animals or monocrotaline- (MCT-) treated rats with saline vehicle or modulated rBMSCs. (b–e) Densitometry of immunoblots showing quantification of changes in CA1, kininogen, SelW, and 14-3-3*η* expression. ^∗^*p* < 0.05; ^∗∗^*p* < 0.01.

The CA1 protein is present in the pulmonary arteries and the epithelium of the alveolar wall (Figure 4(a)). Immunostaining revealed the presence of SelW in the pulmonary artery walls, bronchial walls, and airway epithelium (Figure 4(b)). The expression of CA1 and kininogen increased, and that of SelW and 14-3-3*η* decreased in model groups relative to those in the control groups (Figure 5). Treatment with rBMSC/Cav-1^F92A^ reduced the expression of CA1 and kininogen and upregulated that of SelW and 14-3-3*η*. However, these changes were abolished by treatment with rBMSC/Cav-1^F92A^+L-NAME (Figures 4 and 5).

### 3.5. rBMSC/Cav-1^F92A^ Reactivates the NO Pathway in the Lungs

**Figure 6:**
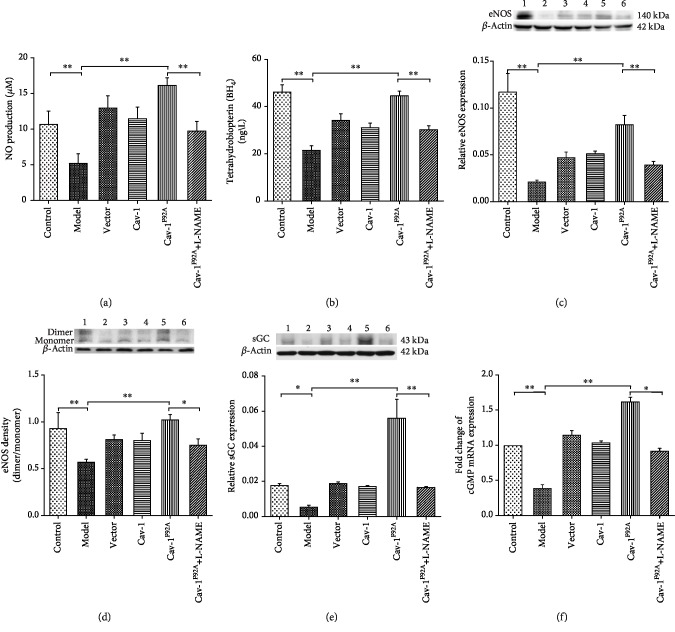
rBMSC/Cav1^F92A^ reactivated NO signal in MCT-induced PAH rats. (a) The changes of NO and BH_4_ production in serum. (c, d) Densitometry of immunoblots showing quantification of changes in eNOS, ratio of eNOS dimerization, and sGC in the lung of control animals or MCT-treated rats with saline vehicle or modulated rBMSCs. (e) Lung cGMP mRNA expression determined by real-time qPCR in control animals or MCT-exposed rats with saline vehicle or modulated rBMSCs. ^∗^*p* < 0.05; ^∗∗^*p* < 0.01.

NO, BH_4_, sGC, eNOS, eNOS dimers and monomers, and cGMP expression are involved in the activation of the NO pathway. Compared with those in the control groups, NO production, BH_4_ production, sGC, eNOS, eNOS dimer/monomer ratio, and cGMP mRNA decreased by approximately 0.5-, 0.47-, 0.17-, 0.36-, 0.77-, and 0.4-fold in MCT-exposed rats, respectively, and were upregulated under rBMSC/Cav-1^F92A^ treatment. However, rBMSC/Cav-1^F92A^+L-NAME treatment attenuated the reactivation of the NO pathway (Figure 6).

## 4. Discussion

NO is a potent cell signal with important and diverse roles in biological processes [19]. The decrease in NO release contributes to the development of PAH [2]. Cav-1^F92A^, which attenuates the inhibition of eNOS activity, enhances the production of NO. Moreover, it has been demonstrated the stimulatory effects of Cav-1^F92A^ on angiogenesis in rBMSCs via improving NO production, which may provide a novel treatment for PAH [17]. We hypothesized that in rats with MCT-induced PAH, the administration of rBMSC/Cav-1^F92A^ may ameliorate vascular resistance and restore pulmonary hemodynamics by inhibiting excessive oxidative stress.

In PAH, excessive oxidative stress may contribute to the proliferation and contraction of smooth muscle cells; these effects result in pulmonary artery stenosis [23]. ROS and GSH are commonly tested oxidative stress indices [22]. A growing body of evidence suggests that MSC therapy exerts antioxidative effects by modulating oxidation and redox homeostasis [24]. Additionally, NO, a potent vasodilator, is mechanistically linked to the pathogenesis of PAH [2]. NO also exerts an antioxidative role in cellular signaling [19, 25]. NO also plays an antioxidative role in cellular signaling [18]. In the present study, we observed that MCT-induced PAH is associated with increased oxidative stress, as demonstrated by the increased production of ROS and the downregulation of GSH expression. By contrast, rBMSC/Cav-1^F92A^ treatment drastically attenuates oxidative stress, increases the production of GSH, and decreases the production of ROS, which is correlated with the production of NO.

To explore the signal transduction event that is involved in the inhibitory effect of rBMSC/Cav-1^F92A^ on oxidative stress, we examined CA1/kininogen and SelW/14-3-3*η* signal transduction. The abnormal expression and activity of CA1, a zinc-containing metalloenzyme that belongs to the carbonic anhydrase family, result in excessive oxidative stress and disrupt redox homeostasis [22]. CA1 participates in ischemic-induced cardiac fibroblast development, hyperglycemia-induced endothelial cell death, cell proliferation, proinflammatory cytokine production, and vascular permeability alterations, which can be suppressed by a CA1 inhibitor [9, 26]. CA1 contributes to vascular injury by activating the plasma kallikrein system [11]. Kininogen, a member of the plasma kallikrein system, contributes to oxidative stress and vascular damage [27, 28]. The upregulation of kininogen production in endothelial cells contributes to pulmonary vascular injury and remodeling in MCT-treated rat lungs [29]. By contrast, SelW performs a vasoprotective function by exerting an antioxidative effect [8]. SelW can activate the 14-3-3 protein, which is involved in redox regulation [12, 30]. In mice, the depletion of the 14-3-3*η* protein causes cardiac dysfunction in the ischemic myocardium; this result demonstrates the protective effect of 14-3-3*η* on adverse cardiac remodeling during pressure-overload-induced heart failure [31]. Therefore, the regulation of CA1/kininogen and SelW/14-3-3*η* signal transduction may provide vascular protection by inhibiting oxidative stress. Our present immunostaining and Western blotting results showed that the presence of CA1 and SelW proteins in rat lungs contribute to the regulation of oxidative stress and vascular damage. Additionally, rBMSC/Cav-1^F92A^ downregulates CA1/kininogen signal transduction and upregulates SelW/14-3-3*η* signal transduction; these effects are correlated with the decrease in oxidative stress. Differential regulation was found for eNOS pathway-related proteins including 14-3-3*η* [32]. However, a few studies showed the relationship between the eNOS/NO pathway and the SelW/14-3-3*η* pathway. Notably, we also found that the eNOS/NO pathway upregulates SelW/14-3-3*η* and downregulates CA1/kininogen signal transduction.

The development of PAH results from alterations in signaling pathways, including the NO pathway. Under physiological conditions, vascular NO that is mainly produced by eNOS stimulates sGC to synthesize cGMP, which performs diverse physiological actions, including vasodilation, vasoprotection, antioxidation, anti-inflammation, and antiproliferation [25, 33–35]. BH_4_ is an eNOS cofactor that is essential for maintaining eNOS coupling in NO production. However, in PAH, once synthesized, NO reacts with excessive ROS to form ONOO^−^, which leads to not only the oxidation of BH_4_ and sGC but also the loss or reduction of BH_4_ and sGC; these effects ultimately result in the disruption of the eNOS dimer and the insensitivity of sGC to NO [36]. Moreover, the disruption of the eNOS dimer, also called eNOS uncoupling or eNOS monomerization, decreases NO production and increases oxidative stress [37]. In the present study, we demonstrated that the downregulation of the NO pathway in MCT-exposed rats is reactivated by the administration of rBMSC/Cav-1^F92A^. The reactivated downregulation of the NO pathway may also be involved in the inhibitory effect of rBMSC/Cav-1^F92A^ on oxidative stress. The upregulation of the NO pathway not only suppresses excessive oxidative stress but also inhibits vasoconstriction and vascular cell proliferation to improve pulmonary hemodynamics and reduce pulmonary vascular resistance.

## 5. Conclusions

Our data provided evidence for the change in CA1/kininogen and SelW/14-3-3*η* signal transduction in MCT-exposed rats. Furthermore, our results suggested that rBMSC/Cav-1^F92A^ may mediate oxidative stress through regulating CA1/kininogen and SelW/14-3-3*η* signal transduction via activating the eNOS/NO/sGC/cGMP pathway. These results indicate that rBMSC/Cav-1^F92A^ may provide novel avenues for the targeted treatment of PAH.

## Data Availability

Data will be available on reasonable request.
